# Ki67 Proliferation Index in Germinal and Non-Germinal Subtypes of Diffuse Large B-Cell Lymphoma

**DOI:** 10.7759/cureus.13120

**Published:** 2021-02-04

**Authors:** Atif A Hashmi, Syeda N Iftikhar, Gul Nargus, Omer Ahmed, Ishaq Azeem Asghar, Umme Aiman Shirazi, Anoshia Afzal, Muhammad Irfan, Javaria Ali

**Affiliations:** 1 Pathology, Liaquat National Hospital and Medical College, Karachi, PAK; 2 Pathology, Khyber Medical University, Peshawar, PAK; 3 Internal Medicine, Liaquat National Hospital and Medical College, Karachi, PAK; 4 Pathology, Ascension St. John Hospital, Detroit, USA; 5 Pathology, University of Oklahoma Health Sciences Center, Oklahoma City, USA; 6 Statistics, Liaquat National Hospital and Medical College, Karachi, PAK

**Keywords:** diffuse large b-cell lymphoma (dlbcl), germinal center b-cell-like (gcb), hans algorithm, ki67 index, proliferative index

## Abstract

Introduction

Diffuse large B-cell lymphoma (DLBCL) is an aggressive B-cell lymphoma. The 2016 World Health Organization (WHO) update on hematopoietic tumors suggested that all DLBCL cases should be subtyped into germinal and non-germinal center phenotypes. Ki67 immunohistochemistry is a maker of cell proliferation and thus is used as a prognostic and predictive marker in various tumors of human body. Only a few studies evaluated the proliferative index of DLBCL subtypes in our population. Therefore, in this study, we evaluated the frequency of subtypes of DLBCL in our population and K67 index in each subtype.

Methods

A retrospective observational study was conducted in the Department of Histopathology, Liaquat National Hospital and Medical College, from January 2018 till December 2020, over a period of three years. A total of 101 cases with a histopathological diagnosis consistent DLBCL were included in the study. Immunohistochemical (IHC) stains CD10, B-cell lymphoma 6 (Bcl-6), and multiple myeloma oncogene 1 (MUM1) were applied for the further sub-categorization of DLBCL into germinal center B-cell-like (GCB) and non-GCB subtypes according to the Hans algorithm. The Ki67 index was interpreted in hot spots of the tumor and reported as an average percentage.

Results

Out of 101 DLBCL cases, 47.5% of DLBCL were GCB, while 52.5% were non-GCB subtypes. Bcl-2, Bcl-6, MUM1, c-Myc, CD10, and CD30 expression were noted in 62.4%, 45.5%, 42.6%, 44.6%, 39.6%, and 7.9% cases, respectively. The mean Ki67 index was 72.94±16.69%. The mean Ki67 index in non-GCB-type DLBCL was 77.67±14.80%, which was significantly higher than the mean Ki67 index in GCB-type DLBCL (67.70±17.22%) with a significant p-value (p=0.002). Cervical lymph node was the most common site of DLBCL, while the stomach was the most common extra-nodal site. A significant association of Ki67 index was noted with subtypes of DLBCL. A higher proportion of non-GCB-type DLBCL exhibited greater than 80% Ki67 index than GCB subtype DLBCL. Moreover, a significant association Ki67 index was noted with c-Myc positivity. A higher proportion of c-Myc-positive DLBCL had greater than 80% Ki67 index.

Conclusion

We found that non-GCB-type DLBCL had a higher Ki67 index than GCB subtype DLBCL, portending a poor prognostic significance of non-GCB subtype of DLBCL. Moreover, c-Myc expression was associated with a higher Ki67 index.

## Introduction

Diffuse large B-cell lymphoma (DLBCL) is the most common non-Hodgkin’s lymphoma (NHL) worldwide. DLBCL is a high-grade BCL and can remain asymptomatic until a late disease stage, and symptoms largely depend upon the site of involvement. DLBCL has a propensity to involve any organ system, and can transform from low-grade BCL. The 2016 World Health Organization (WHO) update on hematopoietic tumors suggested that all DLBCL cases should be subtyped into germinal and non-germinal center phenotypes. The gene expression profiling studies divided DLBCL into germinal center B-cell-like (GCB) and activated B-cell-like (ABC) subtypes. The GCB subtype DLBCL has a better prognosis than ABC subtype DLBCL [1]. Immunohistochemical (IHC) stains CD10, B-cell lymphoma 6 (Bcl-6), and multiple myeloma oncogene 1 (MUM1) serve as surrogate markers for gene profiling. The Hans IHC algorithm is a widely used approach to subtype DLBCL into GCB and non-GCB phenotypes. While the definite classification still rests on molecular-based gene profiling, WHO recognized that gene profiling is not universally accessible and therefore IHC-based categorization is a valid substitute. Ki67 immunohistochemistry is a marker of cell proliferation [2]. It has a wide utility in surgical pathology. Many studies have confirmed the diagnostic and prognostic role of Ki67 index in human cancers [3-5]. On the one hand, a high Ki67 index portends an aggressive nature of a tumor and hence poor prognosis; alternatively, the high proliferative index also leads to a better response to chemotherapy in many tumors. To our knowledge, only a few studies have evaluated the proliferative index of DLBCL subtypes in our population. Therefore, in this study, we evaluated the frequency of subtypes of DLBCL in our population and the Ki67 index in each subtype.

## Materials and methods

**Figure 1:**
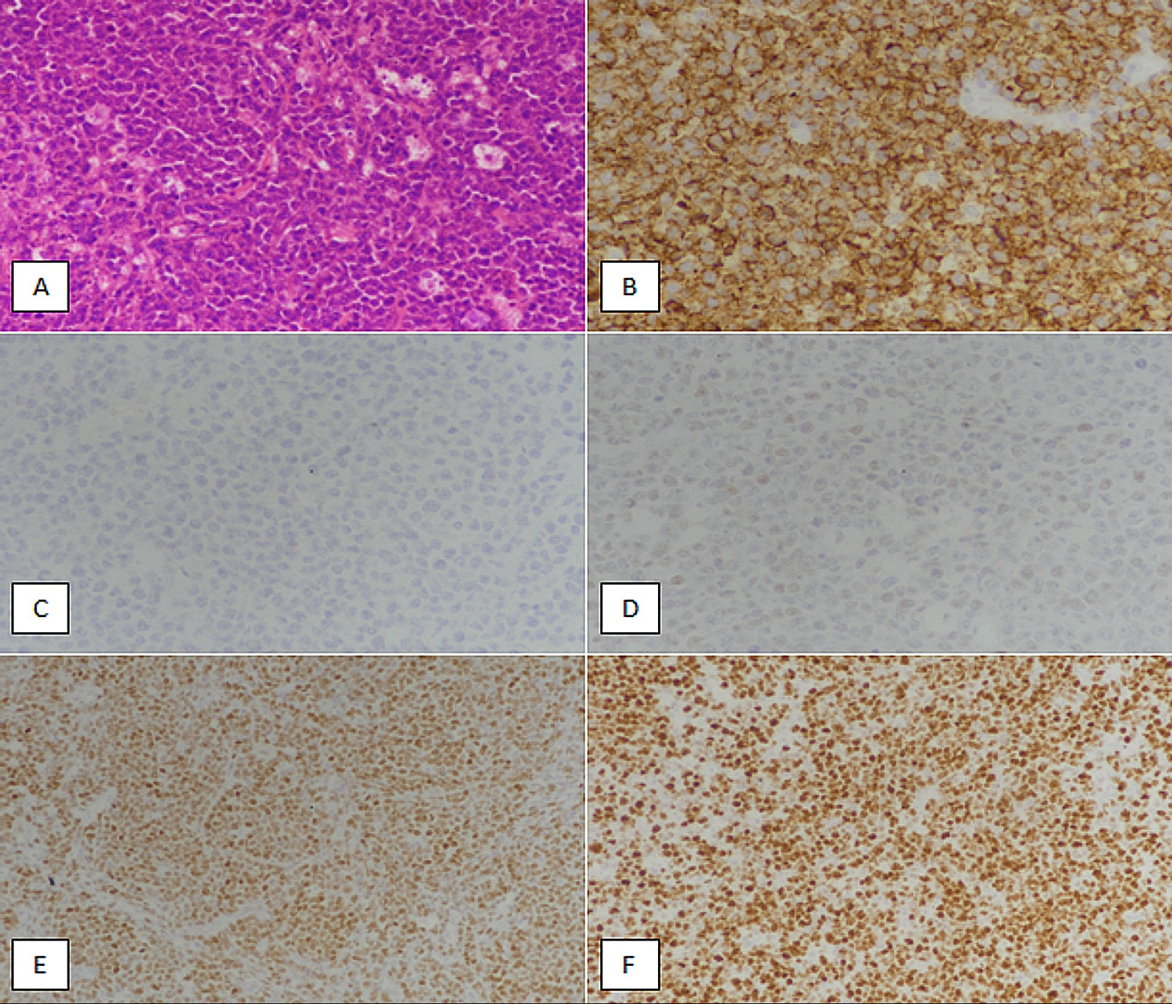
Non-germinal center subtype (non-GCB) diffuse large B-cell lymphoma (A) Hematoxylin and eosin-stained section showing intermediate- to large-sized atypical lymphoid cells with scant cytoplasm arranged in sheets. (B) Tumor cells are diffusely positive (membranous expression) with CD20 IHC stain. (C) Tumor cells are negative with CD10 IHC marker. (D) Bcl-6 IHC staining showing less than 30% weak nuclear expression in tumor cells (more than 30% expression is needed to be interpreted as a positive expression). (E) More than 30% strong nuclear expression is noted with MUM1 IHC marker. (F) Ki67 IHC stain depicting more than 90% proliferative index in tumor cells. IHC, immunohistochemical; Bcl-6, B-cell lymphoma 6; MUM1, multiple myeloma oncogene 1

**Figure 2:**
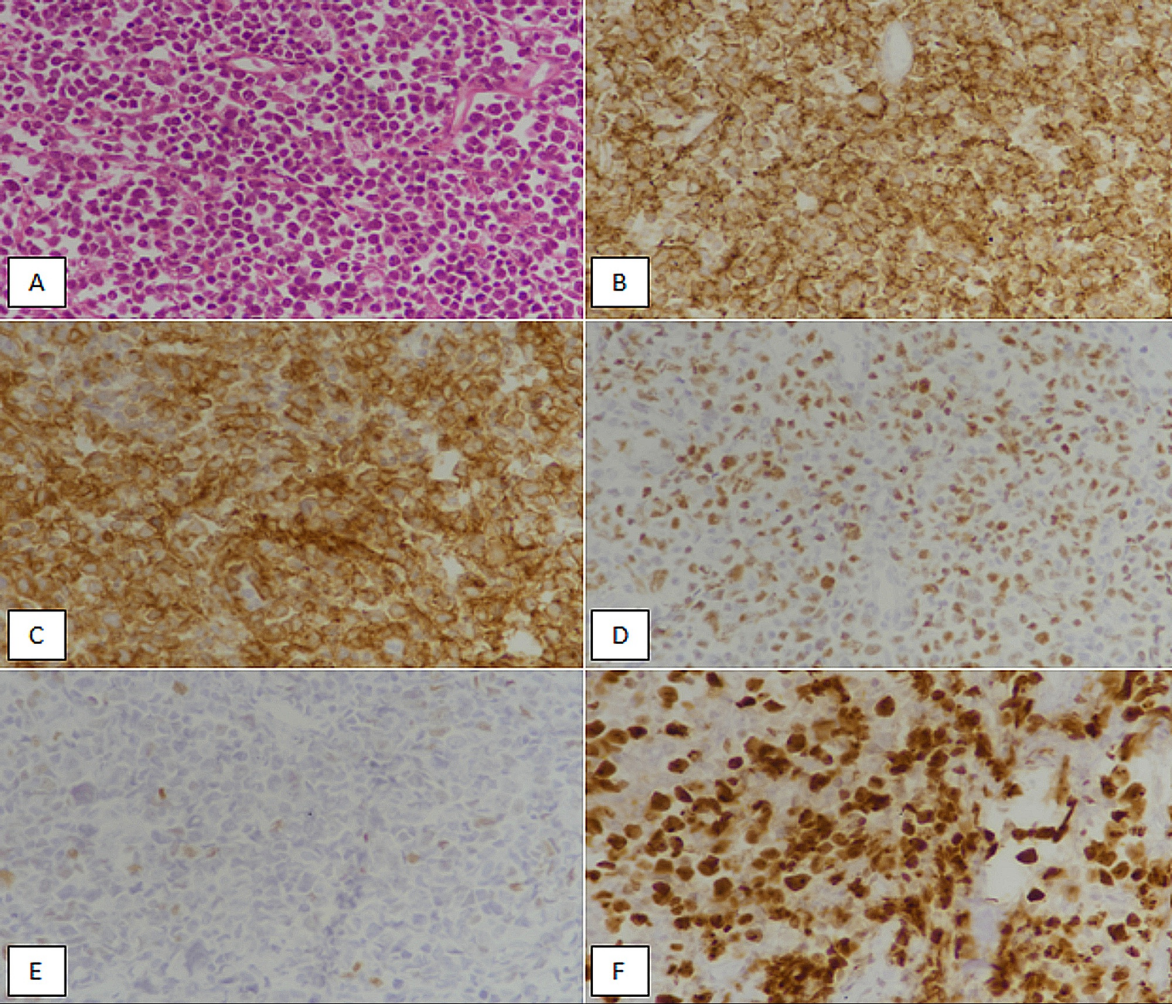
Germinal center subtype (GCB) diffuse large B-cell lymphoma (A) Hematoxylin and eosin-stained section showing large-sized atypical lymphoid cells with scant cytoplasm in a sheet-like architecture. (B) Tumor cells are diffusely positive (membranous expression) with CD20 IHC stain. (C) Tumor cells are diffusely positive (membranous and cytoplasmic expression) with CD10 IHC marker. (D) Bcl-6 IHC staining showing nuclear expression in more than 30% tumor cells. (E) Less than 30% weak nuclear expression is noted with MUM1 IHC marker (more than 30% expression is needed to be interpreted as a positive expression). (F) Ki67 IHC stain depicting 75% proliferative index in tumor cells. IHC, immunohistochemical; Bcl-6, B-cell lymphoma 6; MUM1, multiple myeloma oncogene 1

A retrospective observational study was conducted in the Department of Histopathology, Liaquat National Hospital and Medical College, from January 2018 till December 2020, over a period of three years. All cases were retrieved from the departmental archives. Total 101 cases with a histopathological diagnosis consistent DLBCL were included in the study. DLBCL was diagnosed on the basis of morphology and IHC profile. An IHC panel including CD20, PAX5, CD3, CD5, Tdt, cyclinD1 and CD23 was done to diagnose DLBCL. Moreover, IHC stains CD10, Bcl-6, and MUM1 were applied for further sub-categorization of DLBCL. The Hans algorithm was applied for the subtyping. Cases with CD10 expression of more than 30%, or more than 30% expression of Bcl-6 without MUM1 expression (in the absence of CD10 expression) were classified as GCB subtype DLBCL. All other immunophenotypes were called non-GCB subtype DLBCL. The Ki67 index was interpreted in hot spots of the tumor and reported as an average percentage (Figures 1, 2).

Data analysis was performed using Statistical Package for the Social Sciences, version 26.0 (IBM Corp., Armonk, NY). One-way analysis of variance (ANOVA) and Fisher’s exact test were used to check the association. p-values < 0.05 were considered as significant.

## Results

The mean age of the patients was 52.91±16.71 years and majority of the patients were more than 50 years of age. A total of 47.5% of DLBCL were GCB type, while 52.5% were non-GCB type; 59.4% of specimens were nodal. Bcl-2, Bcl-6, MUM1, c-Myc, CD10, and CD30 expression were noted in 62.4%, 45.5%, 42.6%, 44.6%, 39.6%, and 7.9% cases, respectively. The mean Ki67 index was 72.94±16.69% (Table 1).

**Table 1:** Descriptive statistics of the population under study Bcl, B-cell lymphoma 6; MUM1, multiple myeloma oncogene 1 *Mean±standard deviation

Clinicopathological characteristics	Frequency (%)
Age (years)*	52.91±16.71
Age groups	
≤35 years	18 (17.8)
36-50 years	20 (19.8)
>50 years	63 (62.4)
Gender	
Male	54 (53.5)
Female	47 (46.5)
Subtype	
Germinal center subtype	48 (47.5)
Non-germinal center subtype	53 (52.5)
Site	
Nodal	60 (59.4)
Extra-nodal	41 (40.6)
Specimen type	
Trucut biopsy	46 (45.5)
Excision biopsy	55 (54.5)
Bcl-2	
Positive	63 (62.4)
Negative	38 (37.6)
Bcl-6	
Positive	46 (45.5)
Negative	55 (54.5)
MUM1	
Positive	43 (42.6)
Negative	58 (57.4)
c-Myc	
Positive	45 (44.6)
Negative	56 (55.4)
CD10	
Positive	40 (39.6)
Negative	61 (60.4)
CD30	
Positive	8 (7.9)
Negative	93 (92.1)
Ki67 (%)*	72.94±16.69
Ki67 groups	
≤40%	6 (5.9)
41%-80%	62 (61.4)
>80%	33 (32.7)

The mean Ki67 index in non-GCB-type DLBCL was 77.67±14.80%, which was significantly higher than the mean Ki67 index in GCB-type DLBCL (67.70±17.22%) with a significant p-value (p=0.002).

Cervical lymph node was the most common site of DLBCL, while the stomach was the most common extra-nodal site. No significant association of Ki67 index was noted with the site of DLBCL (Table 2).

**Table 2:** Ki67 index distribution with respect to individual sites of DLBCL DLBCL, diffuse large B-cell lymphoma Fisher’s exact test was applied.

Site of DLBCL	Ki67 index frequency (%)				p-value
	≤40%	41%-80%	>80%	Total	
Retroperitoneal lymph node	1 (16.7)	4 (6.5)	1 (3)	6 (5.9)	0.625
Parotid gland	0 (0)	1 (1.6)	0 (0)	1 (1)	
Mesenteric lymph node	1 (16.7)	4 (6.5)	1 (3)	6 (5.9)	
Cervical lymph node	0 (0)	20 (32.3)	12 (36.4)	32 (31.7)	
Tonsil	0 (0)	2 (3.2)	0 (0)	2 (2)	
Brain	2 (33.3)	5 (8.1)	2 (6.1)	9 (8.9)	
Pancreas	0 (0)	1 (1.6)	0 (0)	1 (1)	
Stomach	1 (16.7)	5 (8.1)	4 (12.1)	10 (9.9)	
Femur	0 (0)	2 (3.2)	0 (0)	2 (2)	
Kidney	0 (0)	1 (1.6)	1 (3)	2 (2)	
Axillary lymph node	1 (16.7)	6 (9.7)	2 (6.1)	9 (8.9)	
Nasopharynx	0 (0)	1 (1.6)	1 (3)	2 (2)	
Liver	0 (0)	2 (3.2)	0 (0)	2 (2)	
Inguinal lymph node	0 (0)	4 (6.5)	3 (9.1)	7 (6.9)	
Large Intestine	0 (0)	2 (3.2)	0 (0)	2 (2)	
Testes	0 (0)	0 (0)	1 (3)	1 (1)	
Ovary	0 (0)	1 (1.6)	0 (0)	1 (1)	
Mediastinal lymph node	0 (0)	0 (0)	2 (6.1)	2 (2)	
Soft palate	0 (0)	0 (0)	1 (3)	1 (1)	
Spleen	0 (0)	0 (0)	1 (3)	1 (1)	
Thyroid	0 (0)	1 (1.6)	1 (3)	2 (2)	

A significant association of the Ki67 index was noted with subtypes of DLBCL. A higher proportion of non-GCB-type DLBCL exhibited greater than 80% Ki67 index than GCB subtype DLBCL (Table 3).

**Table 3:** Association of Ki67 index with subtype of DLBCL DLBCL, diffuse large B-cell lymphoma Fisher’s exact test was applied. *p-value significant as <0.05.

Subtype of DLBCL	Ki67 index			p-value
	Frequency (%)			
	≤40%	41%-80%	>80%	
Germinal center subtype	5 (83.3)	34 (54.8)	9 (27.3)	0.005*
Non-germinal center subtype	1 (16.7)	28 (45.2)	24 (72.7)	

Table 4 shows the association of Ki67 index with age, gender, site of involvement, and IHC expression. A significant association Ki67 index was noted with c-Myc positivity. A higher proportion of c-Myc-positive DLBCL had greater than 80% Ki67 index. On the other hand, no significant association was noted with age, gender, site, or any other IHC marker expression.

**Table 4:** Association of Ki67 index with clinicopathological characteristics and Immunohistochemical expression MUM1, multiple myeloma oncogene 1; Bcl, B-cell lymphoma *Mean±standard deviation; analysis of variance was applied. **Fisher’s exact test was applied. ***p-value significant as <0.05.

Clinicopathological characteristics and Immunohistochemical expression	Ki67 index			p-value
	Frequency (%)			
	≤40%	41%-80%	>80%	
Age (years)*	54.00±14.65	52.96±16.83	52.60±16.13	0.962
Age groups**				
≤35 years	1 (16.7)	12 (19.4)	5 (15.2)	0.896
36-50 years	2 (33.3)	12 (19.4)	6 (18.2)	
>50 years	3 (50)	38 (61.3)	22 (66.7)	
Gender**				
Male	3 (50)	34 (54.8)	17 (51.5)	0.946
Female	3 (50)	28 (45.2)	16 (48.5)	
Site**				
Nodal	3 (50)	38 (61.3)	19 (57.6)	0.843
Extra-nodal	3 (50)	24 (38.7)	14 (42.4)	
Bcl-2**				
Positive	5 (83.3)	37 (59.7)	21 (63.6)	0.625
Negative	1 (16.7)	25 (40.3)	12 (36.4)	
Bcl-6**				
Positive	2 (33.3)	29 (46.8)	15 (45.5)	0.848
Negative	4 (66.7)	33 (53.2)	18 (54.5)	
MUM1**				
Positive	3 (50)	23 (37.1)	17 (51.5)	0.320
Negative	3 (50)	39 (62.9)	16 (48.5)	
C-Myc**				
Positive	1 (16.7)	23 (37.1)	21 (63.6)	0.018***
Negative	5 (83.3)	39 (62.9)	12 (36.4)	
CD10**				
Positive	4 (66.7)	27 (43.5)	9 (27.3)	0.138
Negative	2 (33.3)	35 (56.5)	24 (72.7)	
CD30**				
Positive	0 (0)	6 (9.7)	2 (6.1)	0.825
Negative	6 (100)	56 (90.3)	31 (93.9)	

## Discussion

In this study, we assessed the Ki67 index in subtypes of DLBCL. We noted that non-GCB DLBCL had a higher mean Ki-67 index than GCB-type DLBCL. Moreover, c-Myc expression was associated with a higher Ki67 index.

Among various subtypes of NHL, DLBCL is the most common subtype, accounting for approximately 40% of NHL cases, with some cases having an inferior prognosis than others [6,7]. Ki67 is still considered a useful and reliable marker associated with prognosis, but some studies had mixed results associating high Ki67 expression with the DLBCL outcome and some showed an inverse relationship between the Ki67 index and clinical outcomes [8-11].

Other prognostic factors play an important role in determining the true nature of the behavior of DLBCL, including c-Myc expression, treatment with rituximab, and DLBCL subtype [8,9,11]. Tang et al. compared Ki67 and Bcl-2 as a combined tool to more accurately determine DLBCL prognosis and found it to be superior than using alone [12]. Some studies found Bcl-2 protein and c-Myc co-expression to be an independent risk factor indicating worse outcomes in DLBCL [13,14], but it might be related to the fact that c-Myc-positive DLBCL usually has a high Ki67 index as noted in our study. More studies are therefore required to differentiate among these factors and to delineate the Ki67 relationship with DLBCL prognosis to verify the previous contradicting/conflicting literature [9,12,14,15].

He et al. found that the prognosis varied among different types of DLBCL after the introduction of rituximab and other targeted therapies, which can be important for clinical decision-making and for individualized treatment [16]. Ki67, which is a nuclear non-histone protein and is strictly associated with cell proliferation, is being widely used to monitor numerous malignancies, including lymphoma and neuroendocrine tumors [9,17,18]. Some recent papers have confirmed that a high Ki67 proliferation index is associated with shorter overall survival [19,20]. Consistent with our results, a high Ki67 index in non-GCB type of DLBCL was reported in other studies [9].

We view our study with a few limitations. First, follow-up of the patients was not available to compare survival differences in different subtypes of DLBCL. Clinical data regarding the stage of disease involvement and therapy-related information were not available to evaluate the response to chemotherapy in different Ki67-related subgroups of DLBCL. Therefore, we recommend large-scale clinical studies to assess the follow-up in subgroups of DLBCL based on the Ki67 index. Moreover, molecular studies were not done to evaluate gene rearrangements.

## Conclusions

Subtyping of DLBCL into GCB and non-GCB is considered an essential pathological reporting parameter by WHO. We found a roughly equal proportion of GCB and non-GCB DLBCL in our study. We noted that non-GCB DLBCL had a significantly higher Ki67 index than GCB subtype DLBCL. Moreover, c-Myc expression was also associated with a higher Ki67 index. Ki67 index is a marker of tumor-cell proliferation and thus possesses both prognostic and predictive significance. The high Ki67 index in non-GCB-type DLBCL signifies a poor prognostic feature of this subtype of DLBCL.
